# Phenolic Profile and Biological Evaluation of *Salvia ceratophylloides* Ard.: A Novel Source of Rosmarinic Acid

**DOI:** 10.1002/cbdv.202503276

**Published:** 2026-02-09

**Authors:** Douaa Bekkai, Natalizia Miceli, Maria Fernanda Taviano, Carmelo Coppolino, Francesco Cacciola, Luigi Mondello, Patrizia Trifilò

**Affiliations:** ^1^ Department of Chemical, Biological, Pharmaceutical and Environmental Sciences University of Messina Messina Italy; ^2^ Messina Institute of Technology c/o Department of Chemical, Biological, Pharmaceutical and Environmental Sciences University of Messina Messina Italy; ^3^ Chromaleont s.r.l., c/o Messina Institute of Technology c/o Department of Chemical, Biological, Pharmaceutical and Environmental Sciences University of Messina Messina Italy

**Keywords:** antioxidant activity, chemical diversity, high‐performance liquid chromatography with photodiode array detection and electrospray ionization mass spectrometry [HPLC‐PDA/ESI‐MS], phenolic compounds, *Salvia ceratophylloides*

## Abstract

*Salvia ceratophylloides* Ard. (*Sc*), a rare endemic species from Southern Italy, has been chemically unexplored. We report the first analysis of its polyphenolic profile, antioxidant potential, and preliminary toxicity, compared with the well‐studied *Salvia officinalis* L. (*So*). Plants were cultivated under identical Mediterranean conditions to minimize variability. Leaf hydroalcoholic extracts were examined for polyphenols (high‐performance liquid chromatography with photodiode array detection and electrospray ionization mass spectrometry [HPLC‐PDA/ESI‐MS]), antioxidant activity (2,2‐diphenyl‐1‐picrylhydrazyl [DPPH], reducing power, Fe^2+^ chelation), and toxicity (*Artemia salina* lethality bioassay). Although differing qualitatively, both species showed nearly identical total phenolic content (*So* 274.59 mg/g; *Sc* 274.27 mg/g). Notably, *Sc* was rich in rosmarinic acid and consistently exhibited superior antioxidant activity. It was also nontoxic against *A. salina*, in contrast to *So* (LC_50_ = 79.27 ± 11.62 µg/mL). These findings highlight *Sc* as a promising source of bioactive compounds and warrant further pharmacological and conservation studies.

## Graphical abstract

Among the various extraction techniques investigated, the methanolic ultrasound‐assisted extract (MGME‐UAE) of *Micromeria graeca* L exhibited the strongest bactericidal effect, selectively inhibiting *Proteus mirabilis* (inhibition zone 12.7 ± 0.6 mm; MIC = 6.25 mg/mL). Sagerinic acid (30.37 ± 0.70 mg/g), 5‐caffeoylquinic acid, and caffeic acid, were determined by LC‐PDA/ESI‐MS as the major constituents. MGME‐UAE increased membrane permeability, causing significant leakage of DNA/RNA and proteins.

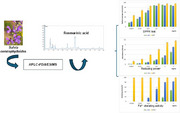

## 1 Introduction

The excessive accumulation of free radicals in the human body is a key factor in the development of degenerative conditions such as chronic inflammation and neurodegenerative disorders [1, 2]. Oxidative stress, which arises from an imbalance between reactive oxygen species (ROS) and the body's antioxidant defense, compromises cellular integrity and normal physiological functions. In this context, plant‐derived polyphenols have attracted considerable attention due to their ability to counteract oxidative damage and to modulate biological processes, including inflammation, cell signaling, and apoptosis [3, 4]. Due to their chemical diversity and multifunctional effects, polyphenols represent an important focus in natural product research. Consequently, the identification of polyphenol‐rich plant species and the evaluation of their chemical composition and biological activity remain central goals in pharmacognosy and phytochemistry.

The genus *Salvia* L. (Lamiaceae), comprising approximately 1000 species distributed across tropical and temperate regions, is among the most diverse genera within the family. In Italy, 20 species contribute significantly to national plant biodiversity. Many *Salvia* species are valued for their culinary, medicinal, and ornamental uses. Among these, *Salvia officinalis* L. (common sage, *So*) is the most extensively studied, with numerous studies reporting antioxidant, anti‐inflammatory, antibacterial, and anticancer properties [5, 6]. Although early investigations primarily focused on its essential oils, increasing attention has been directed toward polar constituents, particularly phenolic acids and flavonoids, as key contributors to its biological activities [7, 8]. In contrast, *Salvia ceratophylloides* Ard. (*Sc*) is a rare and endangered species, endemic to a restricted hilly area near Reggio Calabria in Southern Italy. Despite its ecological significance and distinctive morphological traits, this species has received little attention from a phytochemical and pharmacological perspective. To date, research has mainly addressed its ecophysiology [9, 10] and volatile organic compound profile [11], leaving its phenolic composition and biological potential unexplored. The present study aims to fill this gap by providing the first detailed characterization of the polyphenolic profile, antioxidant activity, and preliminary safety evaluation of *Sc*. A comparative analysis with *So*, used as a reference species, was conducted under controlled cultivation conditions to minimize environmental variability. Beyond its potential biological relevance, *Sc* represents an important component of Southern Italy's endemic flora. Therefore, the characterization of its bioactive compounds contributes not only to natural product research but also to the valorization and conservation of this underexplored species.

## 2 Results and Discussion

### 2.1 Morpho‐Physiological Measurements

According to literature data [9, 10, 11], *Sc* and *So* differed significantly in gas exchange and, consequently, in plant biomass (Table 1).

**TABLE 1:** Mean values ± standard deviation (SD) of total plant biomass (dry weight [DW] plant) recorded at the end of the experimental period (*n* = 5) and of stomatal conductance to water vapor (*g*_L_), transpiration rate (*E*_L_), and photosynthetic rate (An), recorded weekly from February to April in *Salvia ceratophylloides* (*Sc*) and *Salvia officinalis* (*So*).

	*Sc*	*So*	*p* value
**DW plant (g)**	3.62 ± 0.95	5.91 ± 0.73	0.002
***g*_L_ (mmol/m^2^/s)**	314.3 ± 57.2	393.9 ± 65.2	0.006
***E*_L_ (mmol/m^2^/s)**	2.44 ± 0.47	2.24 ± 0.53	0.373
**An (mmol/m^2^/s)**	4.21 ± 1.12	6.71 ± 1.88	0.001

*Note: p* values as resulted by a Student's *t*‐test are reported.

In detail, despite being cultivated under identical optimal conditions, *So* showed higher stomatal conductance and photosynthetic rate than *Sc*, whereas both species showed comparable transpiration rates. Consequently, *So* accumulated significantly greater biomass than *Sc* by the end of the experimental period.

### 2.2 Determination of Polyphenolic Compounds by High‐Performance Liquid Chromatography With Photodiode Array Detection and Electrospray Ionization Mass Spectrometry [HPLC‐PDA/ESI‐MS]

**FIGURE 1:**
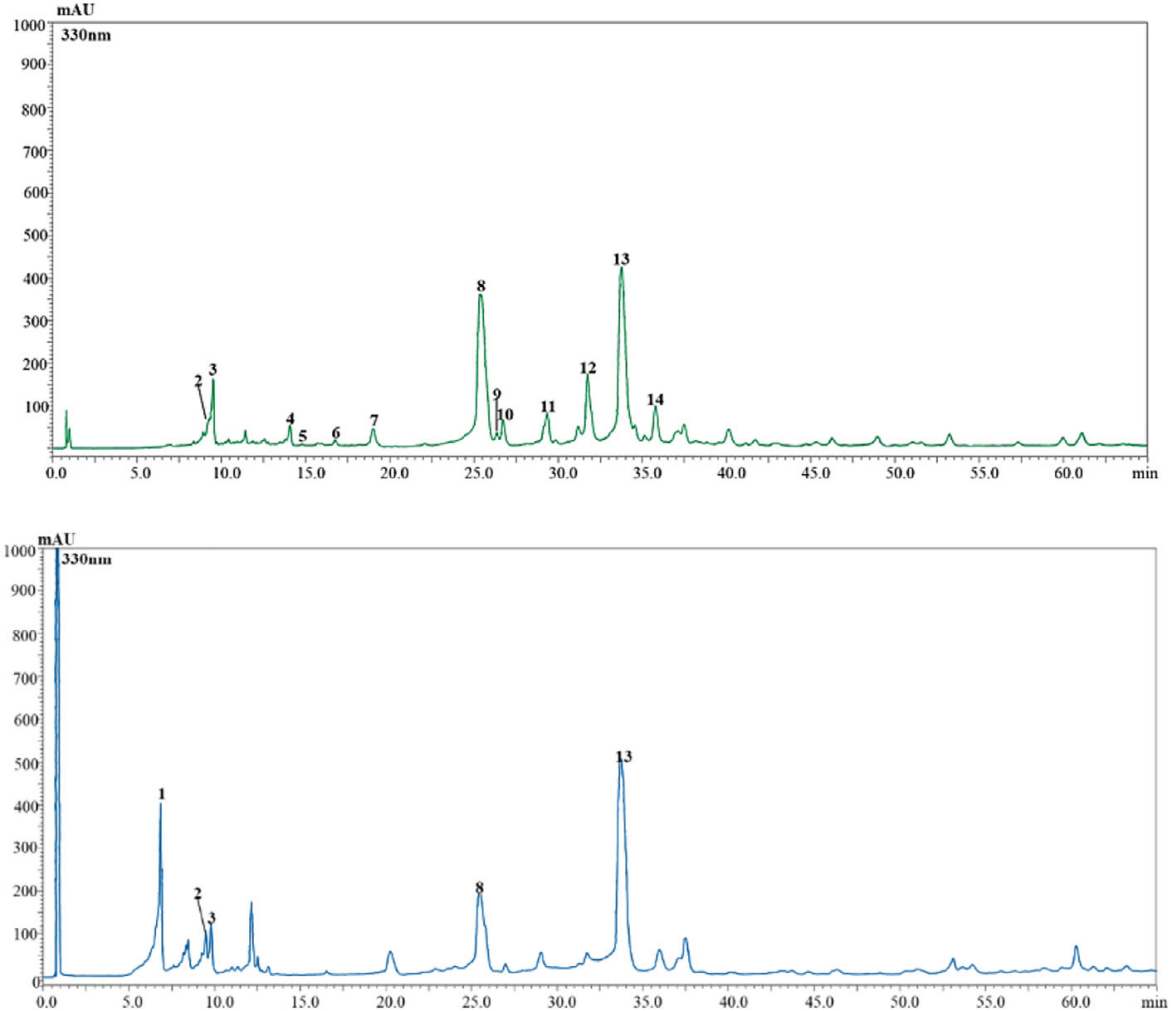
Chromatographic profile of hydroalcoholic extracts from leaves of *Salvia officinalis* (*So*, top) and *Salvia ceratophylloides (Sc*, bottom) at *λ* = 330 nm.

The two *Salvia* species under investigation exhibited distinct qualitative and quantitative phenolic profiles. In total, 14 phenolic compounds were tentatively identified across both extracts: 13 in *So* and 5 in *Sc*. These included seven phenolic acids and seven flavonoids, as illustrated in Figure 1 and detailed in Table 2. Interestingly, despite significant differences in the extraction yields of *Sc* and *So*, approximately double in the former (42%) compared to the latter (23%), as well as in the composition and number of compounds, the polyphenolic amount was remarkably similar between the two species: 274.59 mg/g in *So* and 274.27 mg/g in *Sc*. In *So* extract, rosmarinic acid was the most abundant compound (142.00 ± 3.75 mg/g), followed by luteolin‐7‐*O*‐glucuronide (53.40 ± 3.67 mg/g). In contrast, *Sc* was characterized by higher level of rosmarinic acid (183.18 ± 7.54 mg/g), accompanied by caffeic acid derivative (58.99 ± 1.06 mg/g) as the second most abundant phenolic. This substantial presence of rosmarinic acid in *Sc* suggests that this rare, endemic species may serve as a valuable and novel natural source of this compound, with potential nutraceutical or pharmaceutical applications. To the best of our knowledge, this is the first report on the phenolic composition of the aerial parts of *Sc*. The identification of rosmarinic acid as its dominant phenolic compound is particularly noteworthy, especially considering that *So*, a widely studied member of the Lamiaceae family, is commonly recognized as one of the richest natural sources of rosmarinic acid. When compared with previously published data on other Lamiaceae species, the levels of rosmarinic acid found in *Sc* are especially promising. For instance, *So* has been reported to contain between 12.2 and 296 mg/kg of rosmarinic acid in infusion preparations [12]. In a separate study, Sharma et al. [13] reported concentrations ranging from 67.41 ± 2.03 to 18 821.33 ± 150.20 mg/kg in different extracts (Soxhlet methanol extract, and ultrasound water and methanol extracts) obtained from the leaves of cultivated *So*. These reports, considered within their respective analytical frameworks, indicate that *Salvia* species exhibit a marked capacity for rosmarinic acid accumulation. In this context, the high accumulation observed in *Sc* supports its classification as a particularly rich natural source of this bioactive compound. Phenolic compounds in both species were identified using HPLC‐PDA at 330 nm, based on the comparison of retention times, UV absorption spectra, and mass spectra, with those of available standards or literature data. The predominance of phenolic acids and flavonoids observed here is consistent with chromatographic investigations indicating that these compound classes constitute the principal bioactive fraction in *Salvia* spp. [14]. As expected, our analysis confirmed the presence of rosmarinic acid as the principal phenolic acid in *So*, consistent with numerous previous studies [1, 12, 15, 16].

**TABLE 2:** Characterization of polyphenol compounds in *Salvia officinalis* (*So*) and *Salvia ceratophylloides* (*Sc*) leaf extracts through high‐performance liquid chromatography with photodiode array detection and electrospray ionization mass spectrometry (HPLC‐PDA/ESI‐MS) analysis.

Peak no.	Name	*t*_R_ (min)	UV_max_	[M − H]^−^	*So* mg/g dry extract ± SD	*Sc* mg/g dry extract ± SD	Standard employed for quantification	Refs.
1	Caffeic acid derivative	6.8	326	415, 191	—	58.99 ± 1.06	Caffeic acid	—
2	1‐O‐Caffeolyl glucose	9.3	298sh, 326	341	8.49 ± 0.47	4.29 ± 0.99	Caffeic acid	[18]
3	Caffeic acid	9.4	322	179	19.08 ± 0.87	11.02 ± 0.89	Caffeic acid	[18]
4	Luteolin rutinoside	14.1	270,335	593, 285	1.42 ± 0.08	—	Luteolin‐7‐O‐glucoside	[17]
5	Lithospermic acid A	14.8	327	537	0.19 ± 0.02	—	Caffeic acid	[19]
6	Quercetin 3‐O‐glucuronide	16.8	280sh,343	477, 301	0.75 ± 0.02	—	Quercitrin	[19]
7	Apigenin 7‐O‐Allosyl‐(1‐>2)‐glucoside	19.2	340	593, 269	1.80 ± 0.06	—	Apigenin	[19]
8	Luteolin‐7‐O‐glucuronide	25.6	253, 347	461,285	53.40 ± 3.67	16.79 ± 2.02	Luteolin‐7‐O‐glucoside	[17]
9	Quercetin 3‐O‐glucuronide isomer	26.5	343	477, 301	0.63 ± 0.02	—	Quercitrin	[19]
10	Salvianolic acid C	26.9	344	491	12.46 ± 0.26	—	Salvianolic acid B	[19]
11	Chrysoeriol‐7‐O‐rutinoside	29.3	276, 334	607, 299	5.99 ± 0.06	—	Luteolin‐7‐O‐glucoside	[19]
12	Apigenin‐O‐pentoside	32.0	335	445, 269	7.22 ± 0.32	—	Apigenin	[19]
13	Rosmarinic acid	34.1	328	359, 179	142.00 ± 3.75	183.18 ± 7.54	Caffeic acid	[18]
14	Salvianolic acid k	36.1	287, 323	555	21.16 ± 0.23	—	Salvianolic acid B	[19]
Total					274.59	274.27		

*Note*: Values are expressed as the mean of mg/g of dried extract ± SD, sh: wavelength shoulder.

Qualitatively, our findings are largely consistent with those reported by Martins et al. [17], with a few exceptions. Compounds such as sagecoumarin and sagerinic acid, previously detected in *So*, were not found in our samples.

Notably, significant quantitative differences were also observed, which may be attributed to variations in extraction protocols, environmental growing conditions, or plant phenological stages at the time of sampling. These differences underscore the importance of standardized methods when comparing phytochemical profiles across studies.

Given the comparable phenolic content evidenced for *Sc* and *So*, the much higher amount in the extraction yield of *Sc* appears to be due to other polar compounds belonging to different chemical classes contained in the leaves of *Sc*, extracted with 70% ethanol, which paves the way for further studies aimed at obtaining a thorough characterization of the phytochemistry of this unexplored species.

### 2.3 Antioxidant Activity

**FIGURE 2:**
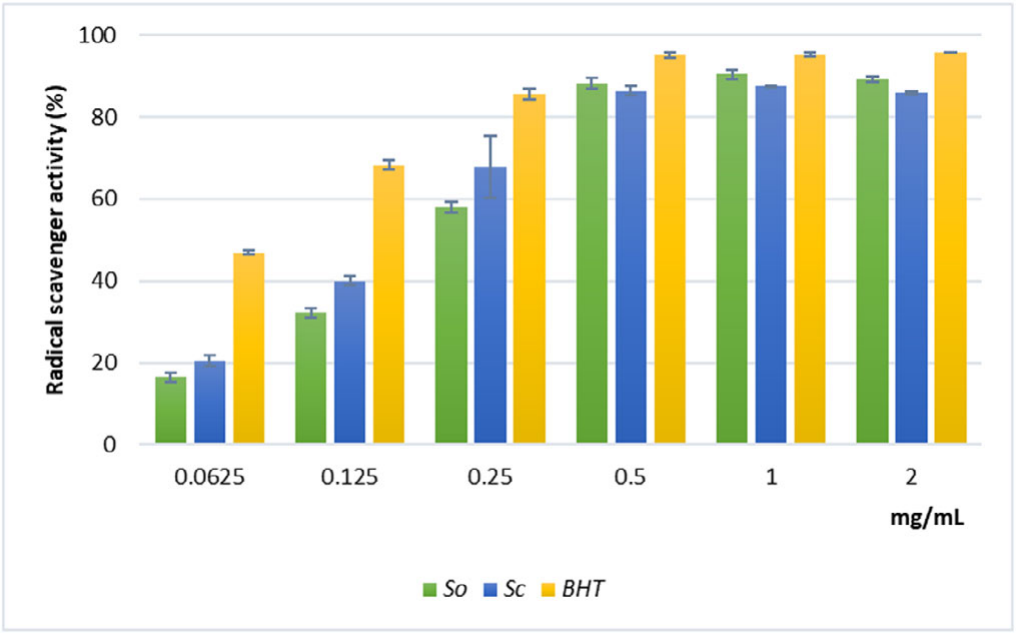
Free radical scavenging activity (DPPH test) of the hydroalcoholic extracts from leaves of *Salvia officinalis* (*So*) and *Salvia ceratophylloides* (*Sc*). The absorbance was recorded after 20 min of incubation at 517 nm. The percentage of scavenging activity was measured as the decrease in absorbance of the samples versus control. Data are expressed as the mean ± SD of three independent experiments (*n* = 3).

Single‐electron transfer (SET) and hydrogen‐atom transfer (HAT) represent two major categories encompassing the primary chemical mechanisms underlying antioxidant activity. The SET mechanism involves the transfer of a single electron to reduce oxidant species. Conversely, the HAT mechanism consists of hydrogen atom donation to neutralize free radicals. Although many antioxidant reactions are characterized as following either SET or HAT pathways, these mechanisms can operate simultaneously. Consequently, the application of multiple complementary assays is recommended to obtain a comprehensive evaluation of the antioxidant properties of plant extracts [20]. In this study, the 2,2‐diphenyl‐1‐picrylhydrazyl (DPPH) test, which involves both HAT and SET mechanisms, and the reducing power assay, based on the SET mechanism, were used to evaluate the primary antioxidant activity of *Sc* and *So* extracts. In addition, ferrous ion chelating activity was used to evaluate the secondary antioxidant capacity. The results of the DPPH test are shown in Figure 2. *Sc* extract exhibited notable radical scavenging activity compared with the standard antioxidant butylated hydroxytoluene (BHT) and higher activity than *So* extract at the lower tested concentrations (0.0625–0.25 mg/mL). Both extracts displayed a similar concentration‐dependent trend, reaching maximum inhibition values of approximately 87% at 0.5 mg/mL. The strong ability of *Sc* to neutralize the stable free radical DPPH demonstrates its potential to mitigate oxidative stress effectively. The IC_50_ value confirmed the higher scavenging activity of the *Sc* extract compared with *So*, although both were less active than BHT (Table 3).

**TABLE 3:** Mean 50% inhibitory concentration (IC_50_) determined by 2,2‐diphenyl‐1‐picrylhydrazyl (DPPH) free radical scavenging activity and by Fe^2+^‐chelating activity assays, and ascorbic acid equivalents (ASE/mL) determined by the reducing power assay of leaf extracts from *Salvia ceratophylloides* (*Sc*) and *Salvia officinalis* (*So*).

	IC_50_, DPPH (mg/mL)	Reducing power (ASE/mL)	IC_50_, Fe^2+^ chelating activity (mg/mL)
*Sc*	0.174 ± 0.007^a^	10.169 ± 0.508	0.471 ± 0.011^a^
*So*	0.225 ± 0.007^b^	9.174 ± 0.186	>2 mg/mL^b^
Standard	BHT	BHT	EDTA
	0.07 ± 0.001	1.443 ± 0.021	0.0067 ± 0.0003
*p* value	<0.001	0.06	<0.0001

*Note*: Data are expressed as the mean ± SD (*n* = 3). Standards: BHT for DPPH and reducing power; EDTA for Fe^2+^ chelation. Differences between *Sc* and *So* were evaluated using Student's *t*‐test. Different letters within the same column indicate statistically significant differences (*p* < 0.05). The corresponding *p* values are also reported.

Abbreviations: BHT, butylated hydroxytoluene; EDTA, ethylenediaminetetraacetic acid disodium salt dihydrate.

**FIGURE 3:**
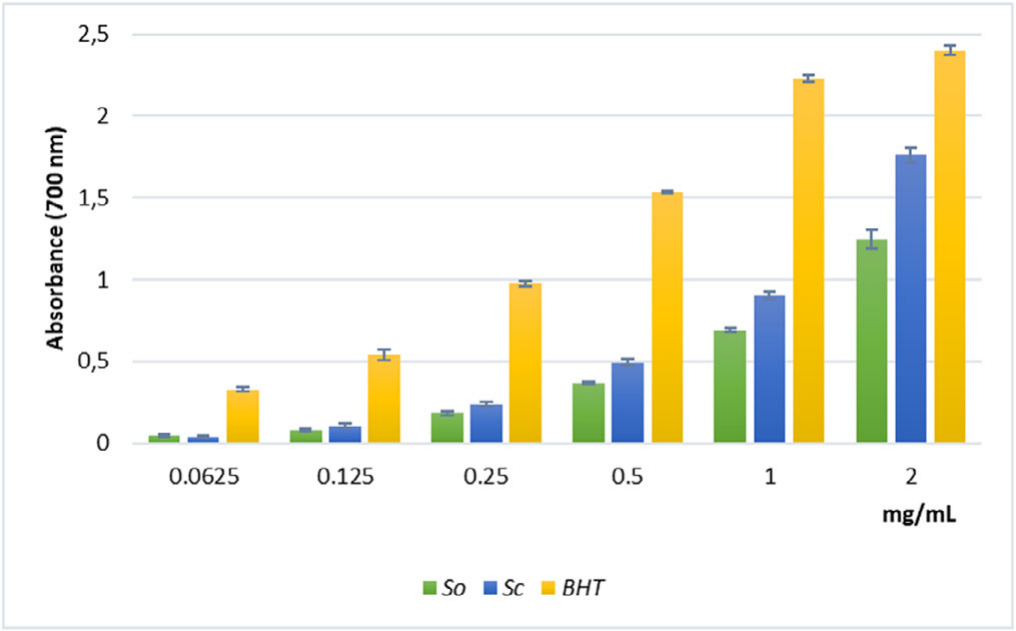
Reducing power of the hydroalcoholic extracts from leaves of *Salvia officinalis* (*So*) and *Salvia ceratophylloides* (*Sc*) evaluated by spectrophotometric detection of Fe^3+^–Fe^2+^ transformation method. The absorbance was recorded after 10 min of incubation at 700 nm. Data are expressed as the mean ± SD of three independent experiments (*n* = 3).

The reducing power assay (Figure 3), which evaluates the ability to interrupt radical chain reaction through Fe^3+^‐Fe^2^^+^ reduction, revealed a moderate but concentration‐dependent activity for the *Sc* extract, reaching an absorbance value of 1.8 at the highest concentration assayed. Nonetheless, the ascorbic acid equivalents per milliliter (ASE/mL) values calculated for the extracts were quite similar (Table 3).

**FIGURE 4:**
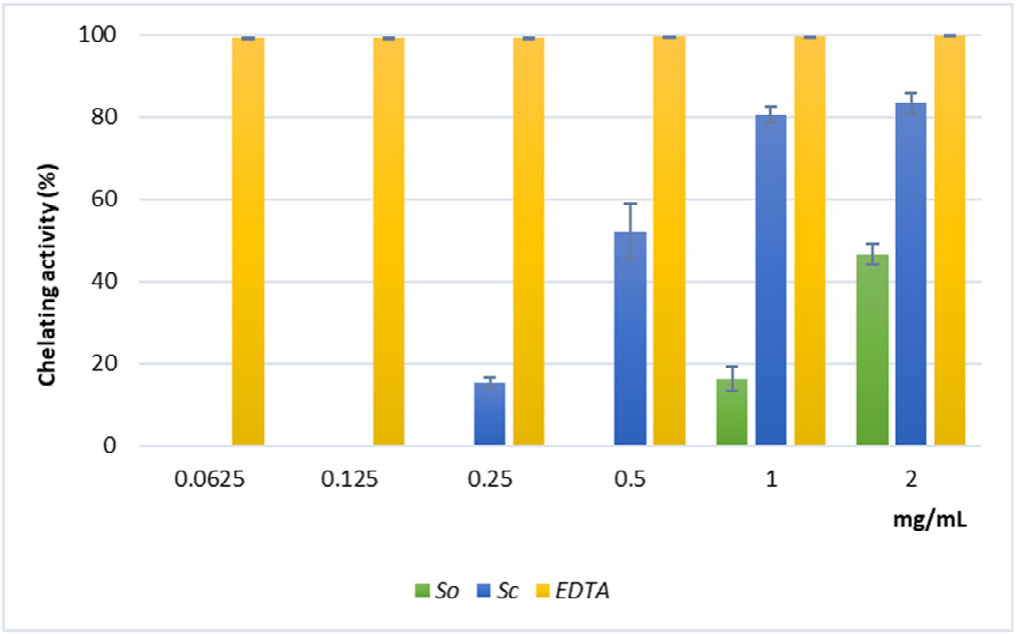
Fe^2+^ chelating activity of hydroalcoholic extracts from leaves of *Salvia officinalis* (*So*) and *Salvia ceratophylloides* (*Sc*). The absorbance was recorded after 10 min of incubation at 562 nm. The percentage of inhibition of ferrozine‐Fe^2+^ complex formation was measured as the decrease in absorbance of the samples versus control. Values are expressed as the mean ± SD of three independent experiments (*n* = 3).

The results of the ferrous ion (Fe^2+^) chelating activity (Figure 4) further indicated a stronger secondary antioxidant capacity for the *Sc* extract compared with *So*. Chelating activity increased with concentration, reaching about 80% at 1 and 2 mg/mL for *Sc*. The IC_50_ value for *Sc* was much lower than that for *So*, although both extracts showed weaker chelating ability than ethylenediaminetetraacetic acid disodium salt dihydrate (EDTA) (Table 3).

Overall, these findings indicated that the *Sc* leaf extract possesses appreciable primary and secondary antioxidant activities under the experimental conditions adopted. These effects may be linked with its higher content of rosmarinic acid, together with caffeic acid and its derivatives, compounds whose antioxidant properties are well documented. Consistent with this interpretation, studies on other medicinal plants have shown that variation in antioxidant performance is largely attributable to differences in the abundance of highly active phenolic acids, particularly rosmarinic acid and structurally related derivatives [21]. Previous investigations have shown the effectiveness of rosmarinic acid in DPPH scavenging, reducing power, and chelation assays [22, 23]. In any case, the possible additive or synergistic effects of the different flavonoid derivatives and phenolic acids contained in the phytocomplexes that may contribute to the observed antioxidant activities should also be taken into account. For example, moderate synergism has previously been demonstrated for a combination of rosmarinic acid and luteolin‐7‐*O*‐glucuronide in the DPPH assay [24]. However, the biological relevance of these antioxidant properties remains preliminary and requires further validation in cellular and in vivo models.

### 2.4 *Artemia salina* Lethality Bioassay

**FIGURE 5:**
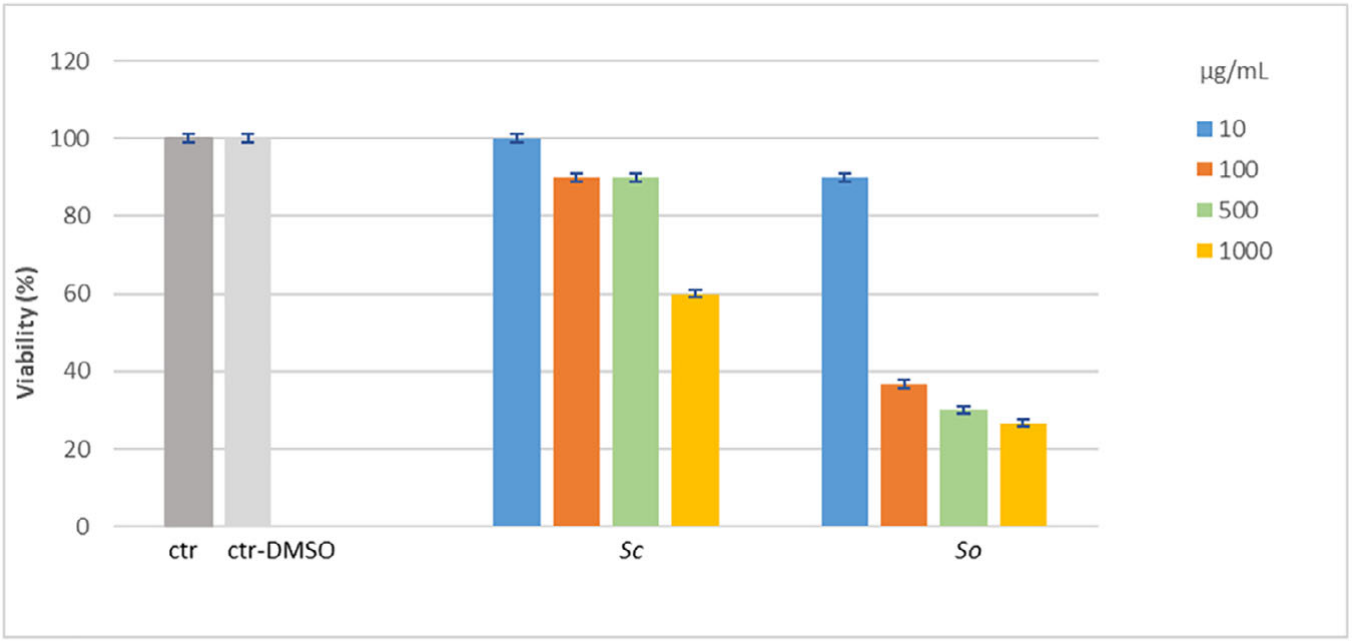
Preliminary toxicity assessment by the *Artemia salina* lethality bioassay of the hydroalcoholic extracts from leaves of *Salvia officinalis* (*So*) and *Salvia ceratophylloides* (*Sc*). The number of surviving nauplii was counted after 24 h treatment, and the vitality percentage was recorded in treated and control groups. ctr: artificial seawater; ctr‐DMSO: artificial seawater added with 2% DMSO. Values are expressed as the mean ± SD of three independent experiments (*n* = 3).

The toxicity of *Sc* and *So* extracts was preliminarily evaluated using the *Artemia salina* lethality bioassay, a widely adopted screening method for assessing the toxicity of plant extracts. This assay offers several advantages, including simplicity, low cost, reproducibility, and ease of implementation, making it suitable for early stage safety evaluation [25]. The results of the assay are shown in Figure 5. According to Clarkson's toxicity criterion, the *Sc* extract was classified as non‐toxic to brine shrimp larvae, as the LC_50_ value was found to be over 1000 µg/mL after 24 h of exposure. In contrast, the *So* extract exhibited marked toxicity, with an LC_50_ value lower than 100 µg/mL, equal to 79.27 ± 11.62 µg/mL. No mortality was observed in the control groups.

The marked toxicity highlighted for *So* is an unexpected result, because some literature data report the safety of *So* preparations. The lack of acute toxicity in rats was shown for an 80% ethanol extract obtained from leaves and flowers of *So* by maceration [26]. Another recent study demonstrated the absence of acute toxicity in mice for a 70% ethanol leaf extract obtained from leaves of *So* by 24‐h maceration [27]. Our disagreeing result could be explained by the selected extraction procedure. Ultrasound‐assisted extraction is an alternative and more efficient extraction method compared to conventional maceration, which can ensure improved recovery of bioactive components. It can be hypothesized that the use of this technique, in combination with a moderate temperature and using 70% ethanol as solvent, led to the extraction of phytoconstituents in high concentration, which may also include toxic compounds, such as the terpenes thujone and camphor [28]. Interestingly, the *Sc* extract obtained with the same procedure was found to be nontoxic; this finding would seem to indicate a lower content of toxic constituents in the leaves of *Sc*. The different toxicity of *Sc* and *So* extracts could be clarified by further phytochemical investigations.

## 3 Conclusion

This study provides the first phytochemical characterization and preliminary biological evaluation of the rare endemic *Sc*. Despite its restricted geographical distribution, this species was found to accumulate high levels of rosmarinic acid and to exhibit notable antioxidant capacity in a set of in vitro assays, together with an absence of toxicity in preliminary *A. salina* bioassay. In comparison, *So* displayed lower antioxidant performance and higher toxicity under the same experimental conditions.

Taken together, these findings suggest that *Sc* is a promising source of bioactive phenolic compounds. However, the biological effects reported here should be regarded as preliminary, as they are based exclusively on in vitro antioxidant assays and a simple toxicity screening model. Further investigations involving cellular systems and in vivo studies are necessary to substantiate its potential relevance for nutraceutical or pharmaceutical applications. Additionally, the results highlight the importance of conserving this underexplored endemic species as part of Italy's phytochemical biodiversity [29].

Future research could also investigate the use of *Sc* extracts as natural antioxidant additives in biodegradable or other eco‐friendly packaging materials. The phenolic compounds may enhance the functional properties of such films, although this remains a preliminary hypothesis requiring experimental validation (e.g., [30, 31]).

## 4 Experimental Section

### 4.1 Plant Material and Growth Conditions

Experiments were performed on 5‐month‐old plants of *Salvia ceratophylloides* Ard. and *Salvia officinalis* L. Seeds of both species, kindly provided by the Botanical Garden of the University of Messina, were soaked in distilled water for 24 h and sown in greenhouse trays in October 2023. One month after germination, individual seedlings were transplanted into 3.5 L plastic pots filled with a nutrient‐rich potting substrate formulated for vegetable cultivation (Comodo, Tercomposti S.P.A., Italy).

Plants were grown in a greenhouse at the Department of Chemical, Biological, Pharmaceutical and Environmental Sciences (ChiBioFarAm), University of Messina. The greenhouse, oriented northwest, received natural daylight supplemented with LED lamps (Led Spectrum Grow 6, I‐GROW s.r.l., Italy), providing a 16 h light/8 h dark photoperiod and a photosynthetic photon flux density of 450 µmol/m^2^/s. Temperature was maintained between 24°C during the day and 18°C at night.

In February 2024, plants were transferred to an open terrace at the ChiBioFarAm Department, where they were exposed to natural environmental conditions and regularly irrigated to maintain field capacity. Leaf sampling for chemical analyses was conducted in April 2024.

### 4.2 Morpho‐Physiological Measurements

To monitor plant physiological status throughout the experiment, stomatal conductance to water vapor (*g*_L_), transpiration rate (*E*_L_), and net photosynthetic rate (An) were measured weekly from February to April. Measurements were performed on at least five fully expanded leaves per species using a portable photosynthesis system (LCi Analyzer, ADC Bioscientific Ltd., Herts, UK).

At the end of the experimental period, plant biomass was assessed. Roots (carefully rinsed to remove soil), stems, leaves, and flowers were oven‐dried at 80°C for 3 days to determine their dry weight (DW).

### 4.3 Extraction Procedure

For the preparation of the extracts, the leaves of *Sc* and *So* were lyophilized, finely ground, and subjected to extraction using 70% ethanol (1:10 w/v). A preliminary maceration at 25°C was performed for 1 h, followed by ultrasonic‐assisted extraction at 50°C for 15 min, repeated twice.

The combined extracts were filtered and concentrated to dryness using a rotary evaporator. Extraction yields were calculated on the basis of 100 g of lyophilized plant material and amounted to 42% for *Sc* and 23% for *So*. The yield obtained for *So* agrees with that previously found by Glisic et al. [32] in the 70% ethanol leaf extract of *So* (1:12 w/v) obtained using ultrasound‐assisted extraction for 45 min at 40°C, resulting in approximately 20%.

### 4.4 Determination of Polyphenolic Compounds by HPLC‐PDA/ESI‐MS

The qualitative and quantitative characterization of the polyphenolic components of *Sc* and *So* extracts was performed using HPLC‐PDA/ESI‐MS.

The dried extracts were redissolved in 70% ethanol and filtered through a 0.45 µm Acrodisc nylon membrane filter (Merck Life Science, Merck KGaA, Darmstadt, Germany) before analysis. LC–MS grade solvents, including water, acetonitrile, and formic acid, along with analytical standards (caffeic acid, apigenin, salvianolic acid B, luteolin‐7‐*O*‐glucoside, and quercitrin), were also purchased from Merck Life Science, Merck KGaA, Darmstadt, Germany.

Chromatographic analysis was carried out using a Shimadzu HPLC system (Kyoto, Japan) equipped with a CBM‐20A controller, two LC‐30AD dual‐plunger parallel‐flow pumps, a DGU20A3R degasser, a CTO‐20AC column oven, a SIL‐30AC autosampler, an SPD‐M30A photodiode array (PDA) detector, and an LCMS‐2020 mass spectrometer. The system employed an ESI source operating in negative ionization mode. Separations were performed on an Ascentis Express C18 column (150 × 2.1 mm; 2.7 µm) obtained from Merck Life Science, Merck KGaA, Darmstadt, Germany [33].

The mobile phase consisted of water (solvent A) and acetonitrile (solvent B), both acidified with 0.1% formic acid (v/v), under the following gradient elution conditions: 0 min: 0% B—10 min: 10% B—20 min: 11% B—30 min: 15% B—50 min: 18% B—65 min: 23% B.

The flow rate was set at 0.5 mL/min, and the injection volume was 2 µL. PDA detection was applied over the 190–450 nm range and monitored at 330 nm (sampling frequency: 40 Hz, time constant: 0.050 s).

Mass spectrometry (MS) conditions were as follows: scan range: *m*/*z* 100–1200; scan speed: 7500 amu/s; event time: 0.3 s; nebulizing gas (N_2_) flow rate: 1.5 L/min; drying gas (N_2_) flow rate: 15 L/min; interface temperature: 350°C; heat block temperature: 300°C; desolvation line temperature: 300°C; desolvation line voltage: 1 V; interface voltage: −4.5 kV.

Quantification of the polyphenolic content in the extracts was based on calibration curves constructed for five standards: caffeic acid, apigenin, salvianolic acid B, luteolin‐7‐*O*‐glucoside, and quercitrin (Table 4). Data acquisition and processing were performed using Shimadzu LabSolution software version 5.97.

**TABLE 4:** Regression equations, correlation coefficients (*R* ^2^), LoD and LoQ, repeatability, and accuracy values for each reference material employed.

Compounds name	Calibration curve	*R* ^2^	LoD	LoQ	Repeatability (%) 100 mg/L	Accuracy (%) 100 mg/L
Caffeic acid	y = 4068.7x + 19686	0.9996	0.053	0.161	0.25	100.30
Apigenin	y = 22357x ‐ 1527.1	0.9999	0.037	0.111	0.26	100.50
Salvianolic acid B	y = 3670.4x + 1415.2	0.9999	0.145	0.439	0.14	100.82
Luteolin‐7‐O‐glucoside	y = 12881x + 54935	0.9995	0.048	0.016	0.31	99.58
Quercitrin	y = 8054x + 27465	0.9997	0.034	0.103	0.12	99.85

### 4.5 Antioxidant Activity

To comprehensively assess the antioxidant capacity of the extracts, three in vitro assays were performed: (i) DPPH radical scavenging assay, (ii) reducing power assay, and (iii) ferrous ion chelating activity assay.

4.6

#### 4.6.1 DPPH Radical Scavenging Assay

The DPPH test was carried out to estimate the free radical scavenging activity of *Sc* and *So* hydroalcoholic extracts, using the method reported by Ohnishi et al. [34]. The extracts were tested at concentrations ranging from 0.0625 to 2 mg/mL. For each sample, 0.5 mL of the extract solution was mixed with 3 mL of freshly prepared 0.1 mM DPPH methanolic solution. The mixture was incubated in the dark at room temperature for 20 min, after which the absorbance was recorded at 517 nm using a UV‐1601 spectrophotometer (Shimadzu). BHT was used as the reference standard. Results were expressed as the mean radical scavenging activity (%) ± standard deviation (SD) and the mean 50% inhibitory concentration (IC_50_) ± SD, derived from three independent experiments.

#### 4.6.2 Reducing Power Assay

The reducing power of *Sc* and *So* extracts was determined using the Fe^3+^–Fe^2+^ transformation method, as described by Oyaizu et al. [35]. Extract concentrations ranged from 0.0625 to 2 mg/mL, with BHT and ascorbic acid serving as reference standards. To perform the assay, a reaction mixture was prepared by combining 2.5 mL of 0.2 M phosphate buffer (pH 6.6), 2.5 mL of 1% potassium ferricyanide, and 1 mL of the extract solution. The mixture was incubated at 50°C for 20 min and then rapidly cooled. Subsequently, 2.5 mL of 10% trichloroacetic acid was added, and the mixture was centrifuged at 3000 rpm for 10 min at 4°C. The supernatant (2.5 mL) was combined with 2.5 mL of distilled water and 0.5 mL of 0.1% ferric chloride. The reaction mixture was incubated in the dark at room temperature for 10 min, and the absorbance was measured at 700 nm. Results are presented as mean absorbance values ± SD and ASE/mL ± SD, based on three independent experiments.

#### 4.6.3 Ferrous Ion (Fe^2+^) Chelating Activity Assay

The Fe^2+^ chelating activities of Sc and So extracts were determined spectrophotometrically by measuring the formation of the Fe^2+^‐ferrozine complex, following the protocol described by Kumar et al. [36]. Extract concentrations ranged from 0.0625 to 2 mg/mL, with EDTA as the reference standard. In this assay, 0.05 mL of 2 mM ferrous chloride solution was mixed with 1 mL of the extract solution and 0.5 mL of methanol. Then, 0.2 mL of 5 mM ferrozine solution was added, and the mixture was shaken vigorously before being incubated in the dark at room temperature for 10 min. The absorbance was recorded at 562 nm using a spectrophotometer. Results are reported as the mean inhibition of the ferrozine‐Fe^2+^ complex formation (%) ± SD and IC_50_ ± SD, based on the average of three independent experiments.

### 4.7 *Artemia salina* Leach (Brine Shrimp) Lethality Bioassay

The potential toxicity of *Sc* and *So* extracts was determined using the brine shrimp lethality bioassay [37]. Brine shrimp eggs were incubated under a 60 W lamp that provided direct light and warmth (24–26°C) in artificial seawater (33 g sea salt/L deionized water). To achieve final concentrations in the range of 10–1000 µg/mL, 10 brine shrimp larvae were incubated for 24 h at 24–26°C in artificial seawater mixed with varying volumes of the extracts dissolved in DMSO. Control groups included artificial seawater alone and artificial seawater added with 2% DMSO, corresponding to the highest solvent concentration used in the assay. After counting the number of surviving nauplii, GraphPad Prism 10 was used to calculate the median lethal concentration (LC_50_) values. The test was performed in triplicate. The extracts’ level of toxicity was evaluated using Clarkson's toxicity criterion, which states that an extract is nontoxic if its LC_50_ is greater than 1000 µg/mL [38].

### 4.8 Statistical Analysis

Statistical analyses were performed using SigmaStat 12.0 (SPSS Inc., Chicago, IL, USA). Differences between species in *g*_L_, *E*_L_, An (*n* = 40), DW (five plants per species), and antioxidant assay results (three independent extracts per species, *n* = 3) were assessed using Student's *t*‐test. Data are expressed as mean ± SD, and differences were considered statistically significant at *p* < 0.05.

## Author Contributions

**Douaa Bekkai**: conceptualization, investigation, data curation, writing – original draft. **Natalizia Miceli**: investigation, data curation, writing – original draft. **Maria Fernanda Taviano**: investigation. **Carmelo Coppolino**: investigation. **Francesco Cacciola**: conceptualization, supervision. **Luigi Mondello**: conceptualization. **Patrizia Trifilò**: conceptualization, supervision.

## Conflicts of Interest

The authors declare no conflicts of interest.

## Data Availability

The data that support the findings of this study are available from the corresponding author upon reasonable request.
